# Persistent Ischemia‐Like Electrocardiographic Changes in a Young Breast Cancer Patient on Trastuzumab: A Case Report

**DOI:** 10.1002/cnr2.70619

**Published:** 2026-07-15

**Authors:** Azin Alizadehasl, Mahsa Elahi, Saba Zangeneh, Niloofar Ahmadloo, Mehdi Dehghani, Masoud Sayad

**Affiliations:** ^1^ Cardio‐Oncology Research Center Rajaie Cardiovascular Medical and Research Center, Iran University of Medical Sciences Tehran Iran; ^2^ Radiation Oncology Department, Namazi Hospital Shiraz University of Medical Sciences Shiraz Iran; ^3^ Noncommunicable Diseases Research Center Fasa University of Medical Sciences Fasa Iran; ^4^ Hematology Research Center, Department of Hematology and Medical Oncology, Namazi Hospital Shiraz University of Medical Sciences Shiraz Iran; ^5^ Department of Cardio‐Oncology Kowsar Hospital Shiraz Iran

**Keywords:** cardiotoxicity, case report, electrocardiography, HER2‐positive breast neoplasms, trastuzumab

## Abstract

**Background:**

Trastuzumab has changed the landscape of treatment for HER2‐positive breast cancer, offering a meaningful survival benefit. However, alongside its effectiveness, concerns remain about its potential impact on the heart, particularly when patients develop unexpected ECG changes during therapy without any obvious cardiac symptoms.

**Case Presentation:**

A 34‐year‐old woman receiving trastuzumab was referred to a cardiologist after routine ECG monitoring revealed ST‐segment coving and T‐wave inversions in the precordial leads. She remained asymptomatic, and her cardiac enzymes were within the normal range. Echocardiography showed preserved left ventricular systolic function, and coronary CT angiography revealed no obstructive disease. Despite the persistent ECG findings, she completed her full trastuzumab course uneventfully, with ongoing cardiology follow‐up.

**Conclusion:**

This case highlights how crucial it is to interpret ECG changes during trastuzumab therapy within the broader clinical context. Although the findings may mimic ischemia, they can appear without any actual cardiac symptoms or injury. Recognizing these as potential effects of the medication helps avoid unnecessary testing and treatment interruptions. With careful monitoring and coordination between the oncology and cardiology teams, patients can continue therapy safely, even when ECG abnormalities persist.

**Abbreviations**
ATP: adenosine triphosphate
BCL: B‐cell lymphoma
CCTA: coronary computed tomography angiography
CT: computed tomography
CTRCD: cancer therapy‐related cardiac dysfunction
ECG: electrocardiogram
GLS: global longitudinal strain
LV: left ventricle
LVEF: left ventricular ejection fraction
MI: myocardial infarction
ST: ST‐segment
TTE: transthoracic echocardiogram

## 1 Introduction

The introduction of targeted therapies has transformed cancer treatment, with trastuzumab (Herceptin) playing a key role in improving outcomes for patients with HER2‐positive cancers. Trastuzumab is a humanized monoclonal antibody that selectively binds to the human epidermal growth factor receptor 2 (HER2), a protein overexpressed in certain cancers, including breast and gastric cancers, where it drives tumor growth and progression. By inhibiting HER2 signaling, trastuzumab suppresses cancer cell proliferation and enhances the efficacy of chemotherapy, leading to significant improvements in overall survival and reduced recurrence rates across different stages of HER2‐positive malignancies. The use of this drug is expanding daily, establishing its crucial role in the treatment of various HER2‐positive cancers [1, 2].

Despite its substantial clinical advantages, trastuzumab is associated with potential risks, the most concerning being cardiotoxicity [3]. This typically presents as left ventricular dysfunction and heart failure, necessitating ongoing cardiac monitoring throughout treatment [4]. While reductions in left ventricular ejection fraction (LVEF) are well‐documented, growing evidence suggests that electrocardiographic (ECG) changes, including ST‐segment deviations and T‐wave abnormalities, may also signal trastuzumab‐induced cardiac effects. However, these ECG alterations are not yet well understood and have not been widely adopted in routine clinical practice [5].

Although trastuzumab‐related cardiotoxicity is often reversible, growing reports describe persistent ECG abnormalities that mimic myocardial infarction (MI) in the absence of clinical symptoms, troponin elevation, or obstructive coronary artery disease [6]. These ECG findings, including ST‐segment elevation, T‐wave inversions, and new‐onset bundle branch blocks, pose diagnostic challenges and may lead to unnecessary interventions [5, 6].

In this report, we present the case of a 34‐year‐old woman undergoing trastuzumab therapy who developed persistent ST‐segment coving and T‐wave inversions in the precordial leads, despite normal cardiac biomarkers and imaging findings. Early identification of trastuzumab‐related ECG abnormalities is vital to avoiding misdiagnosis and ensuring effective patient care. The best strategy to reduce cardiotoxicity involves early monitoring and initiating preventive treatment at the right time.

## 2 Case Presentation

A 34‐year‐old woman with HER2‐positive breast cancer, who was undergoing trastuzumab therapy at Motahari Clinic, Shiraz, Iran, presented for routine cardio‐oncology follow‐up after completing her seventh cycle. She had no prior medical history of cardiovascular disease, including hypertension, diabetes, dyslipidemia, or arrhythmias. There was no family history of premature cardiovascular disease, sudden cardiac death, or inherited arrhythmias. She was a lifelong non‐smoker, with no history of alcohol or illicit drug use, and had not received prior chest radiation.

Her treatment regimen included 4 cycles of anthracycline (doxorubicin) and cyclophosphamide, followed by 4 cycles of docetaxel (Taxotere) and trastuzumab. Following the completion of anthracycline therapy, a baseline cardiac evaluation was performed; the ECG remained stable with no new abnormalities, and the LVEF was preserved at 55%. After a total of 8 chemotherapy cycles, the patient underwent surgery and then continued trastuzumab therapy as a single‐agent targeted therapy. Trastuzumab was administered once every 3 weeks. Baseline cardiovascular and metabolic laboratory findings prior to trastuzumab initiation are summarized in Table 1.

**TABLE 1:** Baseline cardiovascular and metabolic laboratory results before starting trastuzumab treatment.

Test	Result	Unit	Reference range
Cardiac troponin I	0.1	ng/ml	< 0.2
Fasting blood sugar (FBS)	99	mg/dL	70–99
Triglycerides	111	mg/dL	< 150
Total cholesterol	173	mg/dL	< 200
HDL	42	mg/dL	30–85
LDL	105	mg/dL	< 130

During routine monitoring after several cycles of trastuzumab, the patient developed ST‐segment coving and T‐wave inversion in the precordial leads, suggestive of myocardial ischemia, which led to her referral to our cardio‐oncology team at Kowsar Hospital, Shiraz, Iran (May 2024). Transthoracic echocardiography (TTE) demonstrated preserved LVEF of 55% and GLS of (−20%). Cardiac magnetic resonance imaging was not performed due to the patient's clinical stability, preserved ventricular function, negative cardiac biomarkers, and low clinical suspicion for myocarditis based on echocardiographic and coronary CTA findings. Echocardiographic surveillance was initiated at close intervals, with assessments performed approximately every month during the early phase of cardiotoxicity monitoring. High‐sensitivity troponin assays were persistently negative, and repeat ECGs showed no dynamic changes. The patient remained entirely asymptomatic, denying chest pain, dyspnea, palpitations, dizziness, syncope, orthopnea, or peripheral edema. Vital signs were stable, and the cardiopulmonary examination was unremarkable.

About 1 month later, GLS declined slightly to −19% (EF 50%–55%). This slight decline in GLS occurred prior to any measurable change in LVEF, indicating subclinical myocardial involvement. This led to initiation of bisoprolol (2.5 mg daily) and rosuvastatin (10 mg daily), while trastuzumab therapy continued. The initiation of cardioprotective therapy was primarily driven by the decline in GLS as an early marker of subclinical cancer therapy–related cardiac dysfunction, rather than ECG abnormalities alone, although the persistent ECG changes further supported close cardiac monitoring. Bisoprolol was preferred for its cardioprotective profile and potential to mitigate early myocardial strain as well as to manage the observed ECG changes; ACE inhibitors were not used because the patient's blood pressure and LVEF were stable [7]. Statin therapy was added to further reduce the risk of cardiac dysfunction [8]. This management approach was consistent with contemporary cardio‐oncology principles, which support early initiation of cardioprotective therapy in patients with subclinical myocardial dysfunction detected by GLS, even in the presence of preserved LVEF. Despite therapy, the ECG changes persisted, prompting coronary computed tomography angiography (CCTA) in August 2024, which revealed normal coronary arteries without evidence of atherosclerosis, plaque, or anomalies. Follow‐up echocardiography confirmed preserved LVEF (55%) and normal diastolic function (*E*/*A* > 1, septal *e*′ 7 cm/s).

By December 2024, the patient reached the lowest point of subclinical myocardial strain, with GLS of (−16.2%) and preserved LVEF of 55%. She continued cardioprotective therapy and regular monitoring, with follow‐up echocardiography thereafter conducted every 3 months, and over the following months, her cardiac function gradually improved. By June 2025, she demonstrated full recovery, with GLS (−24.8%), EF 55%, and normal diastolic parameters (*E*/*A* > 1, septal *e*′ 9 cm/s). She completed all 17 planned cycles of trastuzumab without any adverse cardiac or other events. These persistent ECG changes, in the absence of clinical symptoms or structural cardiac dysfunction, illustrate a potential trastuzumab‐associated myocardial effect that mimics ischemic patterns on ECG but lacks corresponding biochemical or anatomical evidence of ischemia. Serial electrocardiographic changes observed throughout the course of treatment are presented in Figure 1. A chronological summary of the patient's oncologic treatment, cardiac evaluations, and clinical management is provided in Table 2.

**FIGURE 1:**
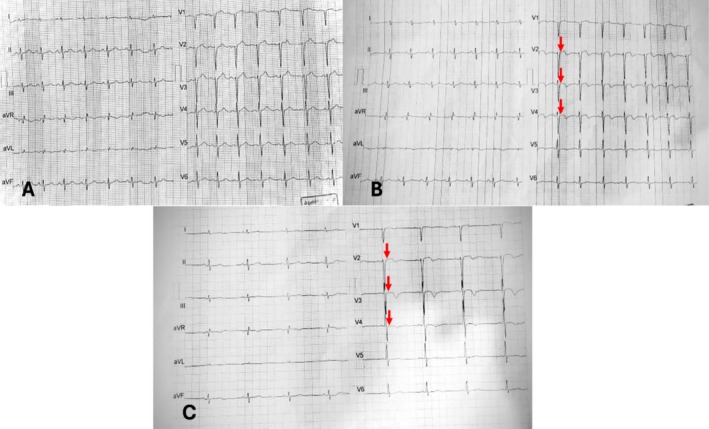
Serial 12‐lead ECGs showing (A) Baseline ECG (pre‐trastuzumab), (B) ST‐segment coving at the J‐point in leads V2 (≈2 mm) and V3 (≈1.5–2 mm) with T‐wave inversion during treatment, and (C) persistent but reduced ST‐segment coving at the J‐point in leads V2–V3 (≈1 mm) with continued T‐wave inversion at 1‐year follow‐up.

**TABLE 2:** Cardio‐oncology timeline summarizing treatment phases and serial cardiac assessments.

Month/year	Clinical event/treatment stage	Cardiac assessment	Intervention/notes
Mid 2023	Start chemotherapy: 4× AC → 4× docetaxel + trastuzumab	Cardiac status stable	—
Early 2024	Breast surgery, continue trastuzumab (single‐agent, q3w)	Cardiac status stable	—
May 2024	Referral to cardio‐oncology team after abnormal ECG during trastuzumab cycles	ECG: ST‐segment coving + T‐wave inversion; LVEF 55%; troponin negative‐GLS (−20%)	Patient asymptomatic
June 2024	Follow‐up	GLS declined slightly to (−19%); (EF 50%–55%)	Start bisoprolol 2.5 mg daily + rosuvastatin 10 mg daily; continue trastuzumab
Aug 2024	Persistent ECG changes	CCTA: normal coronary arteries, LVEF (55%) and normal diastolic function (*E*/*A* > 1, septal *e*′ 7 cm/s)	—
Dec 2024	Subclinical myocardial strain at lowest value	GLS of (−16.2%) and preserved LVEF of (55%)	Continue cardioprotective therapy
Jun 2025	Full recovery	GLS (−24.8%), EF (55%), and normal diastolic parameters (*E*/*A* > 1, septal *e*′ 9 cm/s)	Completed all 17 cycles trastuzumab, no adverse events

## 3 Discussion

In this case, a 34‐year‐old woman with HER2‐positive breast cancer exhibited persistent ischemic‐like ECG changes (ST‐segment coving and T‐wave inversion) during trastuzumab therapy, despite being entirely asymptomatic with consistently negative troponin levels. Unlike most previously reported trastuzumab‐associated ECG abnormalities [6, 9, 10, 11, 12, 13], she showed sustained repolarization changes alongside preserved left ventricular function and normal coronary arteries on CT angiography, highlighting the uniqueness of this presentation. Acute coronary syndrome, myocarditis, and Brugada pattern were considered but excluded based on the absence of symptoms, negative troponins, and normal coronary CTA, pointing to a drug‐induced electrical phenomenon rather than structural or ischemic damage. These findings emphasize the importance of ECG monitoring during trastuzumab therapy, even in asymptomatic patients.

Trastuzumab is a monoclonal antibody targeting the HER2 receptor [14, 15, 16]. While essential for treating HER2‐positive breast cancer [17], it carries a risk of cardiovascular complications, particularly when combined with anthracyclines [3]. These can include decreased heart function and left ventricular dysfunction, known as cancer therapy‐related cardiac dysfunction (CTRCD). Patients may also develop atypical ECG changes, such as T‐wave inversions or ST‐segment deviations, which can mimic ischemic patterns [5]. Although these changes often resolve after discontinuing the drug, cumulative exposure can occasionally lead to more serious complications, including heart failure [6].

The broad clinical range of cardiovascular complications associated with trastuzumab is summarized in Table 3. A comparative review of the literature indicates that ischemic‐like ECG alterations are rarely observed on their own; instead, they typically occur alongside significant functional or clinical decline. For instance, in contrast to the asymptomatic course of our patient, other reports describe severe presentations involving substernal pain, dyspnea, and even systemic neurological or gastrointestinal distress [10, 11, 13, 18, 19]. Furthermore, the preserved systolic function in this case shows a clear difference compared to the significant myocardial failure documented by Ribeiro et al. [6] and Lafaras et al. [10], where LVEF dropped to 15% and 27%, respectively. While several documented cases resulted in irreversible damage or life‐threatening arrhythmias that necessitated cessation of therapy [9, 13], our patient's ability to complete the full 17‐cycle regimen highlights a distinct clinical pattern. This lack of correlation suggests that notable repolarization abnormalities do not necessarily reflect the severity of myocardial injury and may instead represent electrical rather than structural cardiac effects.

**TABLE 3:** ECG abnormalities, clinical symptoms, and cardiac outcomes in trastuzumab‐treated patients.

Authors	Age	Gender	Cancer type	ECG findings	Symptoms	Cardiac function (LVEF)	Outcome/recovery
Olin et al. (2007) [11]	19	Female	HER2+ multifocal breast cancer	Sinus bradycardia, new T‐wave inversions in anterior precordial and lateral leads	Substernal sharp severe chest pain	LVEF: 50%–55%	Partial resolution of ECG changes
Olin et al. (2007) [11]	69	Female	Stage II HER2+ breast cancer	Atrial fibrillation (110 bpm), later accelerated junctional rhythm (75 bpm)	Unwitnessed syncopal event, weak femoral pulse No CHF symptoms No chest pain	LVEF: 39%–45% (stable)	Persistent arrhythmia, intubation, aspiration pneumonia, sepsis
Ribeiro et al. (2012) [6]	69	Female	HER2+ breast cancer	Advanced LBBB, ST‐segment elevation (anterior wall)	Dyspnea	LVEF: 15% → 25% at 30 days	Partial recovery
Hidalgo et al. (2013) [9]	59	Male	Metastatic esophageal adenocarcinoma	New anterolateral T‐wave inversions	Chest tightness, SOB, nausea	40% (new WMA)	Irreversible cardiac damage
Tlegenova et al. (2022) [13]	61	Female	HER2+ breast cancer	Frequent ventricular extrasystoles, unstable ventricular tachycardia	Weakness, headache, dizziness, tachycardia, SOB	LVEF: 69% → 56%	Discharged with improvement; trastuzumab discontinued after 13/18 cycles
Lafaras et al. (2024) [10]	20	Male	Metastatic gastroesophageal junction cancer	Negative T waves and elevated ST segments in leads II, V4, V5, V6	Intermittent precordial chest pain radiating to the scapular regions bilaterally, numbness in the left arm, epigastralgia, nausea, headache, and arthralgia	LVEF: 27% → 62% (recovered in 3 days)	Reversible ischemic dysfunction
Patel et al. (2025) [12]	77	Female	Stage IV breast cancer	Non‐specific ST‐T changes	Right‐sided weakness, facial droop, slurred speech	LVEF: 35%, LV thrombus	LVEF recovered to 60%; LVT resolved after anticoagulation; trastuzumab held indefinitely
Our case (Alizadehasl et al. 2026, [5])	34	Female	HER2+ breast cancer	ST coving, T wave inversion	Asymptomatic	Stable LVEF	No troponin elevation, stable over time

A focused review of the cases summarized in Table 3 indicates that only a limited number of published reports have described ischemia‐like ECG changes in the absence of left ventricular dysfunction, and in most of these cases such abnormalities were either transient or associated with clinical symptoms, biomarker elevation, or reduced ejection fraction. Importantly, continuation and completion of trastuzumab therapy in the presence of persistent ECG abnormalities, as observed in our patient, has been rarely documented in the literature.

It is also important to consider that prior anthracycline exposure may have contributed to a degree of underlying myocardial vulnerability in this patient. Although baseline left ventricular function was preserved and there was no clinical evidence of cardiotoxicity, anthracyclines are well known to cause subtle, subclinical myocardial injury that may not be detected by standard imaging techniques. Accordingly, a potential additive or synergistic cardiotoxic effect between anthracyclines and subsequent trastuzumab therapy cannot be entirely excluded. However, the absence of biomarker elevation, preserved ventricular function, and the isolated persistence of ECG abnormalities in this case make a primary structural form of cardiotoxicity less likely as the dominant mechanism.

Trastuzumab‐induced cardiotoxicity primarily results from HER2 inhibition, a pathway essential for cardiomyocyte survival and stress adaptation [18, 19]. This blockade can disrupt cellular metabolism, causing ATP depletion and altered BCL‐X expression, which directly affect ionic homeostasis and the electrophysiological properties of cardiac cells. ATP depletion may impair the function of membrane Na^+^/K^+^‐ATPase and calcium channels, thereby disturbing the repolarization phase, manifested as ST segment coving and T‐wave inversion on ECG [20, 21, 22]. This process, which can be considered predominantly electrical remodeling, may occur without structural damage or apoptosis, explaining why left ventricular function and biomarkers remained stable in our patient despite the striking ischemia‐mimicking ECG changes [23, 24]. These findings provide a mechanistic link between trastuzumab's molecular effects and its electrophysiologic consequences, demonstrating that ECG abnormalities can occur independently of mechanical injury.

The clinical relevance of trastuzumab‐induced ECG changes lies in their ability to mimic ischemic patterns, despite the absence of myocardial injury or clinical symptoms. These ECG changes could be mistaken for signs of myocardial ischemia, potentially leading to unnecessary diagnostic interventions if not recognized as a trastuzumab side effect [3]. Understanding that trastuzumab can cause such effects is critical to avoiding over‐treatment and reducing unnecessary concerns for both clinicians and patients [25, 26]. In this case, close coordination between oncology and cardiology specialists ensured careful monitoring and management of the patient's cardiac health, allowing her to complete all cycles of trastuzumab without major complications. These findings emphasize the importance of a cardio‐oncology approach with continuous monitoring and interdisciplinary collaboration to reduce cardiovascular risks and improve patient outcomes.

A major strength of this case is the thorough cardiac monitoring, including frequent ECGs, coronary CTA, and serial GLS measurements, which allowed for the detection of subclinical myocardial strain despite a preserved LVEF. However, some limitations should be noted. The absence of cardiac MRI and myocardial biopsy limited further characterization of subclinical myocardial involvement. While treatment was completed safely with cardioprotection, the lack of long‐term follow‐up is a limitation, as the late‐stage cardiovascular impact of these trastuzumab‐induced ECG abnormalities requires more extensive study.

## 4 Conclusion

This case illustrates that persistent ischemic‐like ECG changes during trastuzumab therapy can be associated with a completely benign clinical course and no evidence of myocardial injury, representing the key finding of this report. The main challenge lies in accurately distinguishing these drug‐related abnormalities from actual ischemic events, using careful clinical judgment. The case emphasizes the critical role of clinical context in interpreting such ECG findings. Provided there is no symptom progression, biomarker rise, or drop in LVEF, trastuzumab treatment can safely continue without alteration. A systematic monitoring strategy, involving serial cardiac evaluations and tight collaboration between oncology and cardiology teams, helps maintain treatment safety and avoids unwarranted interruptions.

## Author Contributions

**Mahsa Elahi:** writing – review and editing. **Azin Alizadehasl:** data curation, supervision. **Masoud Sayad:** writing – review and editing, supervision, investigation. **Saba Zangeneh:** writing – original draft, writing – review and editing. **Niloofar Ahmadloo:** data curation, validation. **Mehdi Dehghani:** investigation.

## Funding

The authors have nothing to report.

## Ethics Statement

The study was approved by the Ethics Committee of the Rajaie Cardiovascular Medical and Research Center, Iran University of Medical Sciences. Furthermore, the study was conducted in accordance with the applicable guidelines and regulations, including the Declaration of Helsinki.

## Consent

Written informed consent was obtained from the patient regarding the reporting of her data.

## Conflicts of Interest

The authors declare no conflicts of interest.

## Data Availability

The data that support the findings of this study are available from the corresponding author upon reasonable request.

## References

[1] P. Collier , M. Hussain , Z. B. Popovic , and B. P. Griffin , “Cardiac Surveillance for Anti‐HER2 Chemotherapy,” Cleveland Clinic Journal of Medicine 88, no. 2 (2021): 110–116. 33526465 10.3949/ccjm.88a.19150

[2] A. Grela‐Wojewoda , M. Püsküllüoğlu , B. Sas‐Korczyńska , et al., “Biomarkers of Trastuzumab‐Induced Cardiac Toxicity in HER2‐Positive Breast Cancer Patient Population,” Cancers 14, no. 14 (2022): 3353. 35884413 10.3390/cancers14143353 PMC9313458

[3] G. Piotrowski , R. Gawor , A. Stasiak , Z. Gawor , P. Potemski , and M. Banach , “Cardiac Complications Associated With Trastuzumab in the Setting of Adjuvant Chemotherapy for Breast Cancer Overexpressing Human Epidermal Growth Factor Receptor Type 2–a Prospective Study,” Archives of Medical Science 8, no. 2 (2012): 227–235. 22661994 10.5114/aoms.2012.28549 PMC3361034

[4] D. L. Keefe , “Trastuzumab‐Associated Cardiotoxicity,” Cancer 95, no. 7 (2002): 1592–1600. 12237930 10.1002/cncr.10854

[5] A. Alizadehasl , M. Mohseni , F. N. Bezanjani , M. Maleki , and K. Roudini , “Trastuzumab and ECG Changes Dilemma,” International Journal of Hematology‐Oncology and Stem Cell Research 17, no. 2 (2023): 63. 37637771 10.18502/ijhoscr.v17i2.12641 PMC10452953

[6] K. Ribeiro , C. Miranda , J. M. Andrade , et al., “Trastuzumab‐Induced Myocardiotoxicity Mimicking Acute Coronary Syndrome,” Case Reports in Oncology 5, no. 1 (2012): 125–133. 22666200 10.1159/000337576 PMC3364043

[7] M. Guglin , J. Krischer , R. Tamura , et al., “Randomized Trial of Lisinopril Versus Carvedilol to Prevent Trastuzumab Cardiotoxicity in Patients With Breast Cancer,” Journal of the American College of Cardiology 73, no. 22 (2019): 2859–2868. 31171092 10.1016/j.jacc.2019.03.495 PMC6557296

[8] M. Obasi , A. Abovich , J. B. Vo , et al., “Statins to Mitigate Cardiotoxicity in Cancer Patients Treated With Anthracyclines and/or Trastuzumab: A Systematic Review and Meta‐Analysis,” Cancer Causes & Control 32, no. 12 (2021): 1395–1405. 34406595 10.1007/s10552-021-01487-1 PMC8541988

[9] S. Hidalgo , C. A. Albright , and G. L. Wells , “A Case of Trastuzumab‐Associated Cardiomyopathy Presenting as an Acute Coronary Syndrome: Acute Trastuzumab Cardiotoxicity,” Case Reports in Cardiology 2013, no. 1 (2013): 473979. 24826288 10.1155/2013/473979 PMC4008353

[10] C. Lafaras , K. Lafara , T. Achladas , et al., “Acute Reversible Atypical Takotsubo Cardiomyopathy Following Trastuzumab and 5‐Fluorouracil in a Young Patient,” Cureus 16, no. 10 (2024): e72764, 10.7759/cureus.72764. 39618604 PMC11608011

[11] R. L. Olin , S. S. Desai , K. Fox , and R. Davidson , “Non‐Myopathic Cardiac Events in Two Patients Treated With Trastuzumab,” Breast Journal 13, no. 2 (2007): 211–212. 17319871 10.1111/j.1524-4741.2007.00408.x

[12] V. Patel , T. Okan , J. L. Yee , and J. Patel , “A Cycle of Complications: A Case Report on Trastuzumab‐Induced Takotsubo Cardiomyopathy Causing Left Ventricular Thrombosis Complicated by Thromboembolic Stroke,” European Heart Journal ‐ Case Reports 9, no. 2 (2025): ytaf002. 39935812 10.1093/ehjcr/ytaf002 PMC11811414

[13] Z. Tlegenova , S. Balmagambetova , B. Zholdin , et al., “A First Approach to Identifying Cardiotoxic Effects of Breast Cancer Chemotherapeutic Treatment in Kazakhstan,” Journal of Clinical Medicine of Kazakhstan 19, no. 1 (2022): 28–35.

[14] J. Castaings , “Cardiac Effect of Oncological Treatment in Patients With Breast Cancer Analyzed by Left Ventricle Deformation and Changes on ECG,” 2021.

[15] J. Jiang , B. Liu , and S. S. Hothi , “Herceptin‐Mediated Cardiotoxicity: Assessment by Cardiovascular Magnetic Resonance,” Cardiology Research and Practice 2022, no. 1 (2022): 1910841. 35265371 10.1155/2022/1910841 PMC8898877

[16] M. A. Mourad , A. S. Ebrahim , A. F. Ziady , E. TEZ , and A. MFA , “Chemotherapy‐Induced Cardiotoxic Effect in Breast Cancer Patients Treated With Trastuzumab (Herceptin) by MRI,” Egyptian Journal of Radiology and Nuclear Medicine 54, no. 1 (2023): 207.

[17] H. Tahir , N. Bardia , K. Bath , et al., “Trastuzumab‐Induced Cardiomyopathy and Intermittent Left Bundle Branch Block,” Cardiology Research 10, no. 4 (2019): 230–235. 31413780 10.14740/cr888 PMC6681842

[18] H. Eaton and K. N. Timm , “Mechanisms of Trastuzumab Induced Cardiotoxicity–Is Exercise a Potential Treatment?,” Cardio‐Oncology 9, no. 1 (2023): 22. 37098605 10.1186/s40959-023-00172-3 PMC10127350

[19] Y. Yarden and M. X. Sliwkowski , “Untangling the ErbB Signalling Network,” Nature Reviews. Molecular Cell Biology 2, no. 2 (2001): 127–137. 11252954 10.1038/35052073

[20] A. Bueno‐Orovio , C. Sánchez , E. Pueyo , and B. Rodriguez , “Na/K Pump Regulation of Cardiac Repolarization: Insights From a Systems Biology Approach,” Pflügers Archiv / European Journal of Physiology 466, no. 2 (2014): 183–193. 23674099 10.1007/s00424-013-1293-1

[21] G. Koulaouzidis , A. E. Yung , D. E. Yung , et al., “Conventional Cardiac Risk Factors Associated With Trastuzumab‐Induced Cardiotoxicity in Breast Cancer: Systematic Review and Meta‐Analysis,” Current Problems in Cancer 45, no. 5 (2021): 100723. 33726923 10.1016/j.currproblcancer.2021.100723

[22] Q. Wu , B. Bai , C. Tian , et al., “The Molecular Mechanisms of Cardiotoxicity Induced by HER2, VEGF, and Tyrosine Kinase Inhibitors: An Updated Review,” Cardiovascular Drugs and Therapy 36, no. 3 (2022): 511–524. 33847848 10.1007/s10557-021-07181-3

[23] X. Liang , Y. Wang , X. Yin , et al., “Electrocardiographic Characteristics of Breast Cancer Patients Treated With Chemotherapy,” Cardiology Research and Practice 2020, no. 1 (2020): 6678503. 33376602 10.1155/2020/6678503 PMC7744229

[24] N. Siri‐Angkul , S. C. Chattipakorn , and N. Chattipakorn , “The Mechanistic Insights of the Arrhythmogenic Effect of Trastuzumab,” Biomedicine & Pharmacotherapy 139 (2021): 111620. 33901874 10.1016/j.biopha.2021.111620

[25] A. Elme , T. Saarto , K. J. Tötterman , et al., “Electrocardiography Changes During Adjuvant Breast Cancer Therapy: Incidence and Risk Factors,” Anticancer Research 33, no. 11 (2013): 4933–4939. 24222132

[26] M. Karaca , H. Kocoglu , I. Bilgetekin , et al., “Ventricular Bigeminal Rhythm Associated With Trastuzumab: A Potential Cardiac Side Effect,” Journal of Cancer Research and Therapeutics 14, no. S2 (2018): S536–S537. 29970721 10.4103/0973-1482.183557

